# ERK1/2 and p38 regulate inter-individual variability in ozone-mediated *IL-8* gene expression in primary human bronchial epithelial cells

**DOI:** 10.1038/s41598-018-27662-0

**Published:** 2018-06-20

**Authors:** Emma C. Bowers, Shaun D. McCullough, David S. Morgan, Lisa A. Dailey, David Diaz-Sanchez

**Affiliations:** 1 Curriculum in Toxicology, University of North Carolina – Chapel Hill, Chapel Hill, NC 27599 USA; 2 Environmental Public Health Division, National Health and Environmental Effects Research Laboratory, U.S. Environmental Protection Agency, Research Triangle Park, NC 27711 USA

## Abstract

Inter-individual variability is observed in all biological responses; however this variability is difficult to model and its underlying mechanisms are often poorly understood. This issue currently impedes understanding the health effects of the air pollutant ozone. Ozone produces pulmonary inflammation that is highly variable between individuals; but reproducible within a single individual, indicating undefined susceptibility factors. Studying inter-individual variability is difficult with common experimental models, thus we used primary human bronchial epithelial cells (phBECs) collected from many different donors. These cells were cultured, exposed to ozone, and the gene expression of the pro-inflammatory cytokine *IL-8* was measured. Similar to *in vivo* observations, we found that ozone-mediated IL-8 expression was variable between donors, but reproducible within a given donor. Recent evidence suggests that the MAP kinases ERK1/2 and p38 mediate ozone-induced *IL-8* transcription, thus we hypothesized that differences in their activation may control *IL-8* inter-individual variability. We observed a significant correlation between ERK1/2 phosphorylation and *IL-8* expression, suggesting that ERK1/2 modulates the ozone-mediated *IL-8* response; however, we found that simultaneous inhibition of both kinases was required to achieve the greatest *IL-8* inhibition. We proposed a “dimmer switch” model to explain how the coordinate activity of these kinases regulate differential IL-8 induction.

## Introduction

Inter-individual variability is observed in nearly every biological response; however this variability is difficult to model and its underlying mechanisms are often poorly understood. Currently the majority of mechanistic studies in the biological sciences do not account for response variability, as they often utilize a single cell line or animal strain. As a result, it is difficult to understand the effects of biological exposures and identify mechanisms of susceptibility and resistance. Current methods of assessing inter-individual variability include the assembly of large panels of cell lines or animal strains across which responses can be compared; however, inherent species differences and/or aberrant cellular processes associated with cell line immortalization may reduce applicability to normal human biology. Using panels of primary cells can overcome many of the aforementioned obstacles, as primary cells have more physiologically relevant biology and can be collected from many different individuals. Unfortunately, obtaining primary cells can be difficult and commercial options are often not cost effective. Our lab specializes in the collection and *in vitro* exposure of primary human bronchial epithelial cells (phBECs) and is thus uniquely poised to study a range of physiologically relevant biological responses. In this model, phBECs are collected from the bronchi of human volunteers, cultured at an air liquid interface, exposed to various stimuli, and then mechanisms related to response variability are examined.

Air pollutants are excellent stimuli to investigate inter-individual variability, as they are ubiquitous, affect millions of individuals, have high degree of morbidity and mortality associated with exposure, and can elicit highly variable responses between individuals^1,2^. An air pollutant that is especially useful in this context is ozone. Decades of human clinical exposure studies have demonstrated that ozone exposure results in pulmonary inflammation that can vary by orders of magnitude even in healthy individuals and cannot be faithfully explained by disease state or traditional risk groups^2–4^. Despite this high level of inter-individual variability, the ozone inflammatory response is highly reproducible within an individual^3,5^. This suggests that there are biological factors dictating the ozone response that have yet to be defined.

The release of pro-inflammatory cytokines is a critical component of the ozone pro-inflammatory response. IL-8 is an important pro-inflammatory chemokine that is a hallmark of environmentally-induced airway inflammation^6,7^. Multiple studies have shown that ozone exposure results in the release of IL-8 protein into the airways and that these levels exhibit extensive variation between individuals^8,9^. Epithelial cells are one of the first cells to encounter inhaled pollutants and are a major source of IL-8 following ozone exposure^10–12^; therefore we used the phBEC system to understand the molecular mechanisms driving IL-8 response variability.

Previous studies using a different phBEC model system found that the mitogen-activated protein kinases (MAPKs) are central signaling mediators of ozone-associated *IL-8* expression, rather than the NFκB pathway, which was previously demonstrated in cell lines^13^. Within the MAPK pathway, the activity of extracellular-signal related kinases (ERK) 1 and 2, as well as p38 have an essential role in ozone pro-inflammatory signaling, as inhibiting these signaling pathways diminishes ozone-induced pro-inflammatory gene expression^13^. While we previously identified MAPKs as key mediators of the ozone pro-inflammatory response, the nature of this relationship is only beginning to be characterized.

In the current study we used large panel of donors to study ozone-mediated *IL-8* expression variability in differentiated phBECs. We sought to answer three critical questions: First, we wanted to determine if phBEC responses to *in vitro* ozone exposure were inherent and donor-specific, as has been observed *in vivo*. Second, we wanted to determine whether previous observations regarding the importance of MAPK activation in ozone-mediated IL-8 induction would be upheld in differentiated phBECs, which closely model the airway as they mimic a pseudostratified epithelium containing basal, goblet, and ciliated cells^14^. Third, we questioned whether ERK1/2 and p38 activation are an important mechanism controlling inter-individual variability in *IL-8* expression. This research will help establish whether the ozone/MAPK/*IL-8* pathway can be used to investigate differential responses to ozone exposure, which will ultimately lead to a better understanding of pro-inflammatory response inter-individual variability.

Here we report that the inhibition of both kinases was required for the suppression of *IL-8* expression, which is consistent with previous findings in the polarized phBEC model. *IL-8* transcriptional responses from individual donors were consistent across repeated collections, suggesting that the magnitude of IL-8 induction is an intrinsic property of phBECs and may be donor-specific. We found that MAPK activity differed between cultures with high and low IL-8 transcriptional responses, with highly responsive cultures exhibiting elevated ERK1/2 pathway activation. These results suggest that an individual’s epithelial cells have intrinsic properties that regulate their response to environmental pollutants, and that one mechanism of this programming may be the differential regulation of the ERK1/2 pathway.

## Results

### Donor-to-donor variability in IL-8 mRNA expression in phBECs exposed to ozone *in vitro*

**Figure 1:**
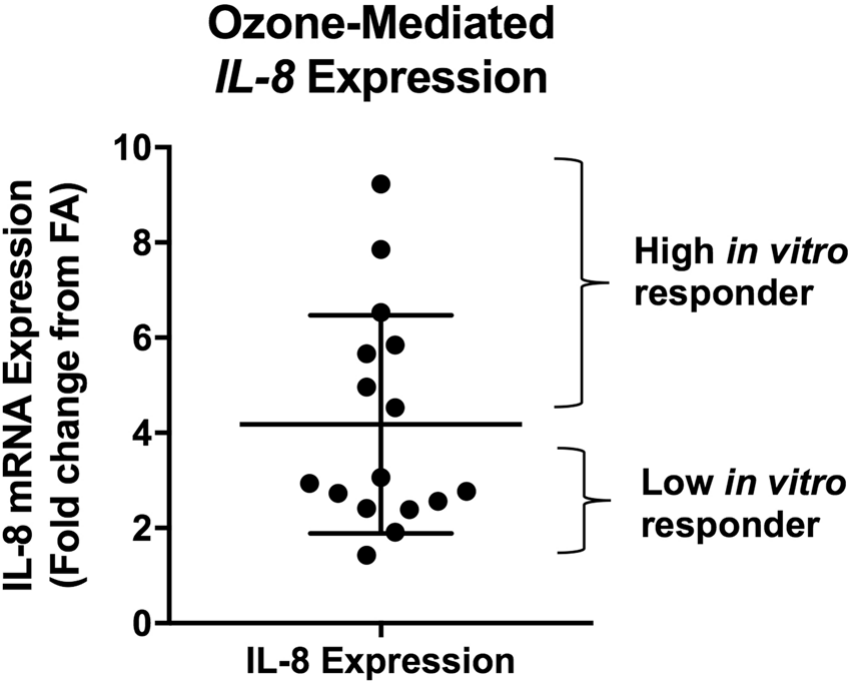
Variability in ozone-mediated *IL-8* induction among phBECs collected from different donors. *IL-8* expression was assessed following *in vitro* exposure of phBECs to ozone (0.5 ppm/2 h). Each data point represents a phBEC culture collected from a different human donor. *IL-8* expression was normalized to β-actin expression and expressed as fold change from filtered air exposure. Mean ± SD shown, n = 16 donors. To further investigate the mechanisms underlying ozone responsiveness using phBECs, we classified cultures as being “high” or “low” responders based on whether *IL-8* inductions fell above or below the group mean (4.2 fold change).

In phBEC cultures exposed to ozone *in vitro* (Fig. 1B), the mean (±SD) *IL-8* induction was 4.18 ± 2.29 fold change from FA. For downstream analysis using the phBEC *in vitro* system, we used the arithmetic mean value of 4.2 fold change from FA to separate donor cultures into “high responders” and “low responders” depending on whether they fell above or below this value. Of the sixteen donors assessed, 7 were high and 9 were low responders.

### Donor-specificity in the IL-8 response

**Figure 2:**
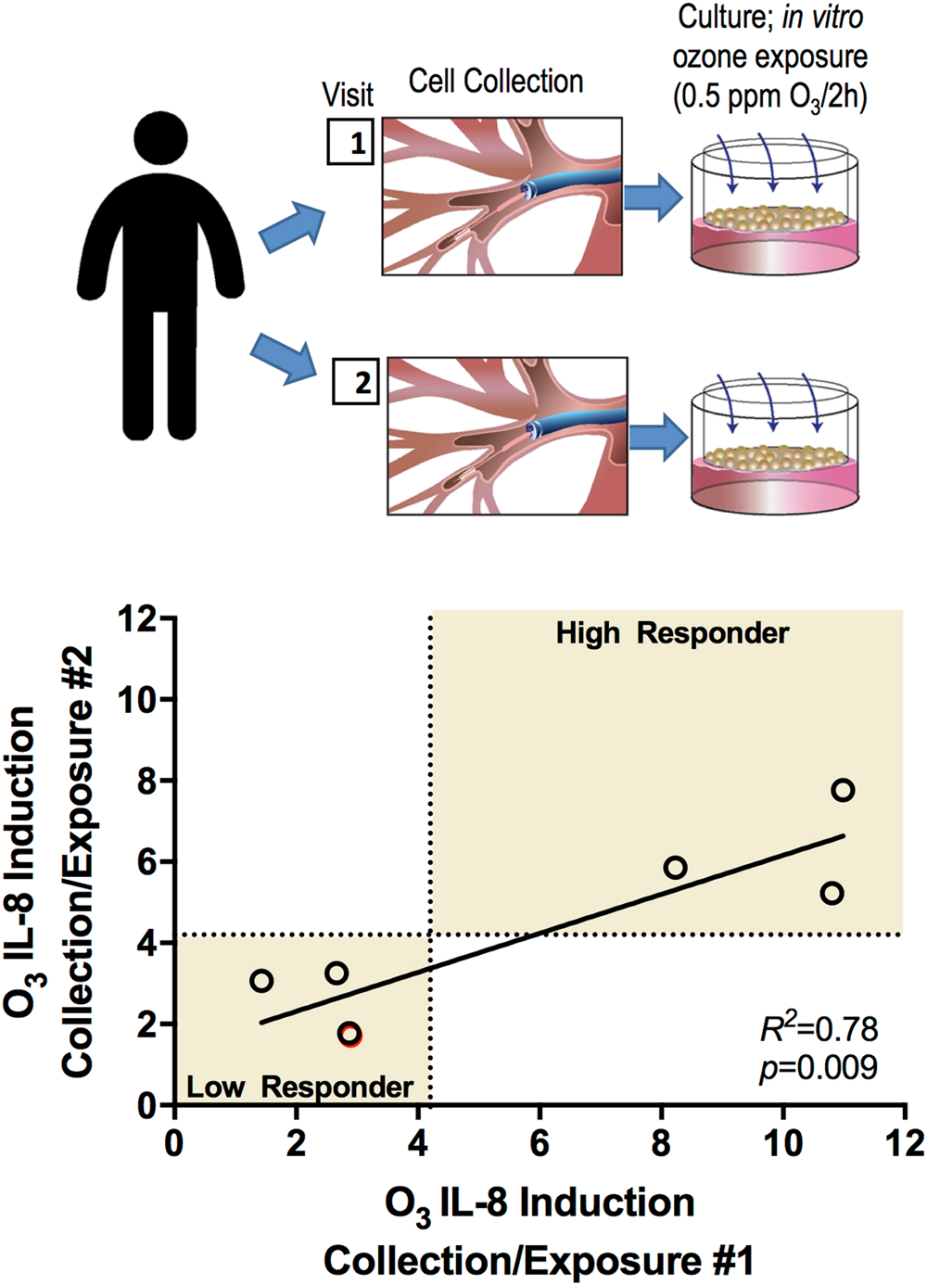
Donor-specificity of phBEC *IL-8* induction following *in vitro* ozone exposure. We investigated whether phBEC cultures collected from the same donors at different times had reproducible *IL-8* expression following ozone exposure. Seven phBEC donors were used with time between collections ranging from 49–453 days. The consistency of ozone response was assessed by comparing the first exposure response (X-axis) with the second exposure response (Y-axis) via linear regression. Dotted lines are drawn at X = 4.2, Y = 4.2, which is the metric by which we differentiated high and low responders. The shaded areas indicate the quadrants the data points would cluster within if cultures were consistent in their response status (high or low) in both collections one and two.

PhBECs collected from the same donor exhibited donor-specificity in ozone associated *IL-8* induction (Fig. 2). Seven phBEC donors had two epithelial cell collections performed. The time between bronchoscopies ranged from 49–453 days. We used the mean described in Fig. 1B, 4.2-fold change from FA, to differentiate high and low responders. For all seven donors, responder status was recapitulated between collections and exposures as indicated by the data points clustering in the shaded quadrants in Fig. 2, as well as strength of linear regression (R^2^ = 0.78, *p* = 0.009). These data suggest that epithelial cell *IL-8* transcriptional responses from specific individuals are reproducible.

### The influence of ERK1/2 and p38 inhibition on the ozone-IL-8 response

**Figure 3:**
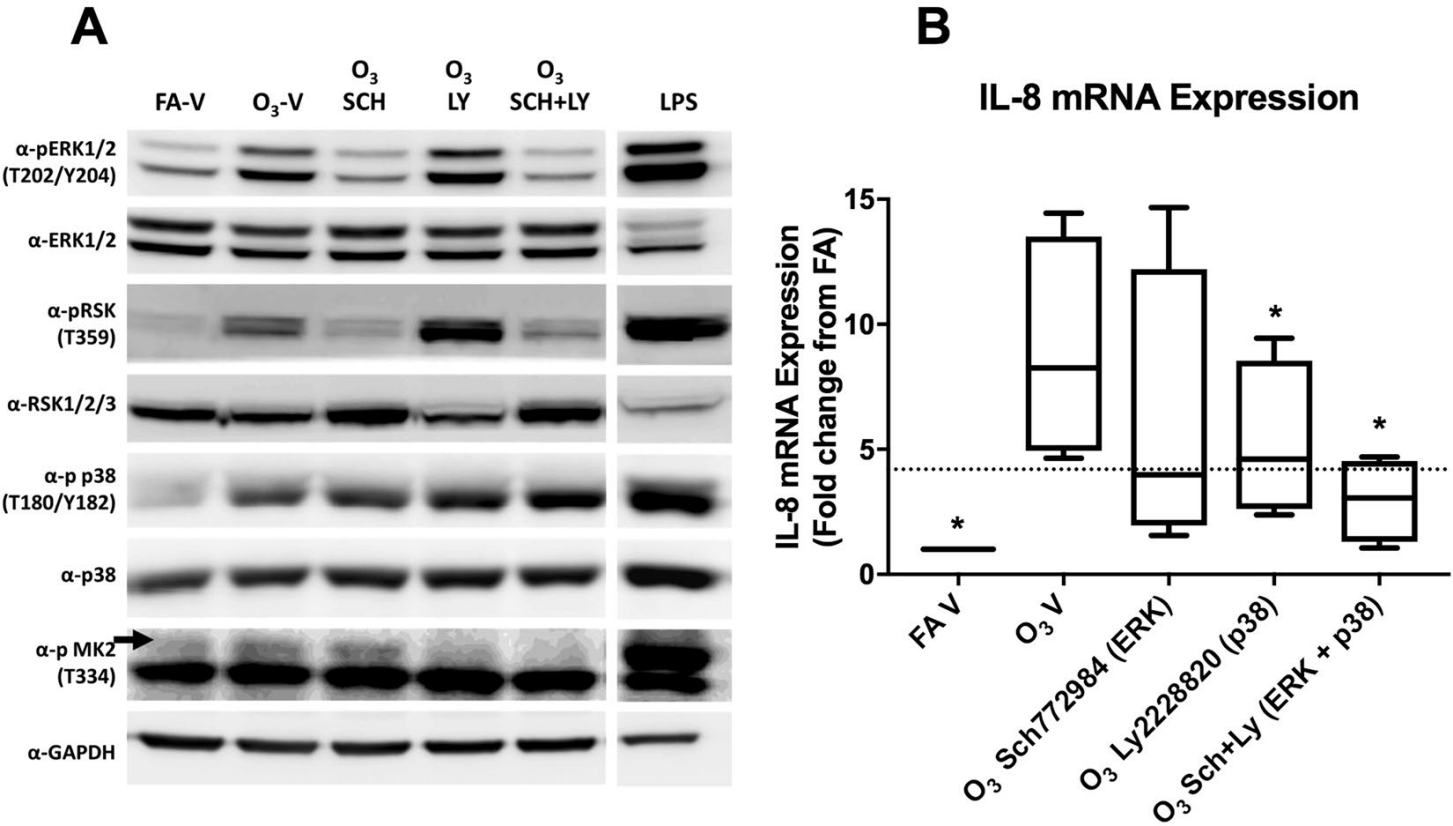
The influence of ERK1/2 and p38 inhibition on ozone-associated *IL-8* induction. To determine whether the activation of the MAP kinases ERK1/2 and p38 were required for ozone-mediated *IL-8* induction, inhibitors of these kinases (250 nM SCH772984 and LY2228820, respectively) were added to cell media two hours prior to ozone exposure. **(A)** The phosphorylation of downstream targets of ERK1/2 and p38, RSK and MK2, respectively, were examined to verify kinase inhibition. Extract from phBECs stimulated with 5 μg/mL LPS for four hours was used as a positive control. The LPS positive control for each mark was run on the same gel, immunoblotted, and imaged at the same time as the blots in the adjacent row. The images were cropped to allow the LPS treatments to be shown in the same lane. (**B**) Seven donors were used for drug treatments, four of which were high responders (shown above) and three low responders (Figure S1). *IL-8* inductions were normalized to a 0.2% DMSO filtered air vehicle control. *IL-8* expression was compared between the ozone-vehicle control (O_3_−V) and all other treatments via 2-way ANOVA. *p < 0.05.

The effect of MAPK inhibition on ozone-associated *IL-8* expression was examined using cells from seven different phBEC donors with unknown responder status. Four of these donors were found to be high *IL-8* responders (Fig. 3) and three were low responders (Figure S1). The addition of inhibitors resulted in the reduced phosphorylation of ERK1/2 and its downstream kinase RSK, as well as MK2, the downstream target of p38 (Fig. 3A). Extract from LPS-stimulated phBECs was used as a positive control for MAPK activation. The LPS positive control for each mark was run on the same gel, immunoblotted, and imaged at the same time as the blots in the adjacent row. The images were adjusted so the LPS treatment could be shown in the same lane. The addition of the ERK1/2 and p38 inhibitors SCH772984 and LY2228820, respectively, resulted in the reduced induction of *IL-8* expression (Fig. 3B). In *in vitro* high responders a statistically significant reduction from O_3_−V levels was observed with the inhibition of p38 and the simultaneous inhibition of both kinases (ANOVA with Dunn’s Multiple Comparisons *p* = 0.008). Mean *IL-8* induction (±SD) for the O_3_−V treatment was 8.26 ± 4.5, which was reduced to 5.26 ± 3.15 following the LY2228820 treatment, and further reduced to 2.96 ± 1.69 following the SCH772984+ LY2228820 treatment. Three low *in vitro* responders also received the inhibitor treatment (Figure S1) but no statistically significant changes in *IL-8* expression were observed.

### Comparison of MAPK phosphorylation in high and low responding cultures

To test the hypothesis that MAPK phosphorylation can explain the inter-individual variability in the *IL-8* ozone response, we compared ozone-associated *IL-8* induction with the activation of the ERK1/2 and p38 pathways following ozone exposure. Using the same cutoff of 4.2-fold change to differentiate high and low *in vitro IL-8* responders, we obtained matched RIPA extracts and RNA samples from six high- and six low-responding phBEC cultures. Ozone-mediated *IL-8* induction in these donors ranged from a 1.43–4.06 fold change in low responders and 4.53 to 14.45-fold change in high responders.

**Figure 4:**
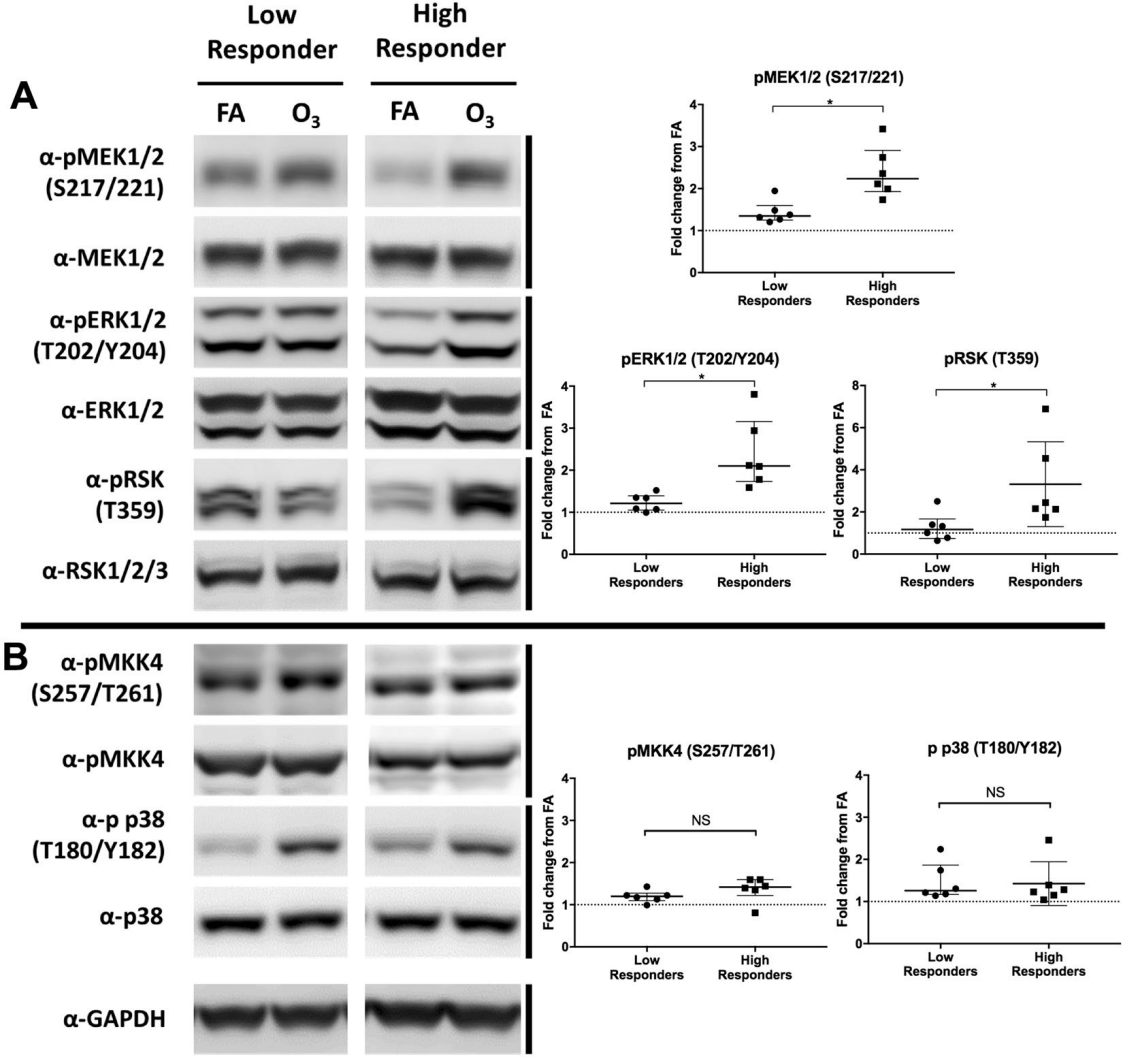
The activation of ERK1/2, p38, and associated kinases in high and low-responding phBEC cultures. Cells were considered “high responders” if their *IL-8* induction was above the group mean (4.2 fold change) and low responders were below this group mean. Protein was collected from twelve donors (n = 12), six high and six low responders. **(A)** Blots from two representative donors (one high, one low) show the phosphorylation of ERK1/2 and an upstream (MEK1/2) and a downstream (RSK) kinase, as well as p38 and its upstream kinase (MKK4). Both sets of blots were run on the same gel, immunoblotted, and imaged at the same time. **(B)** Densitometry analysis was used to calculate the fold change in activation (normalized to filtered air control) for each donor. Median activation and interquartile range are shown for each group. Differences between high and low responders were determined via Mann-Whitney test. *p < 0.05.

A non-parametric Mann-Whitney test was used to assess MAPK phosphorylation differences (Fig. 4) between high and low responders, as several distributions were skewed. Following ozone exposure, the mean fold change from FA (±SD) of ERK1/2 phosphorylation (Fig. 4A) in low responders was 1.23 ± 0.21 and 2.39 ± 0.84 in high responders. After finding a significant difference in ERK1/2 phosphorylation between these groups (*p* = 0.022), we then examined the phosphorylation of the kinases upstream (MEK1/2) and downstream (RSK) from ERK1/2. MEK1/2 phosphorylation in low responders and high responders was found to be significantly different, with low responders having a mean of 1.43 ± 0.27 and high responders a mean of 2.39 ± 0.61 (*p* = 0.004). RSK phosphorylation was also significantly different between these groups, with low responders having a mean of 1.28 ± 0.67 and high responders having a mean of 3.32 ± 2.01 (*p* = 0.026).

After determining that high responders had elevated ERK1/2 pathway activation, we then assessed the phosphorylation of p38 (Fig. 4B). Low responders had a mean p38 phosphorylation of 1.47 ± 0.44 and high responders had a mean of 1.43 ± 0.52. The phosphorylation of p38 was not found to differ between these groups (*p* = 0.94). As an additional check of the p38 pathway, we also examined the phosphorylation of MKK4, which is a MAP kinase kinase upstream of p38 that is activated by ozone exposure^13^. Low responders had a mean MKK4 phosphorylation of 1.20 ± 0.14 and were not significantly different from high responders, which had a mean of 1.37 ± 0.29 (*p* = 0.13). This indicated that the activation of the p38 pathway does not differ between high and low *in vitro* *IL-8* responders.

**Figure 5:**
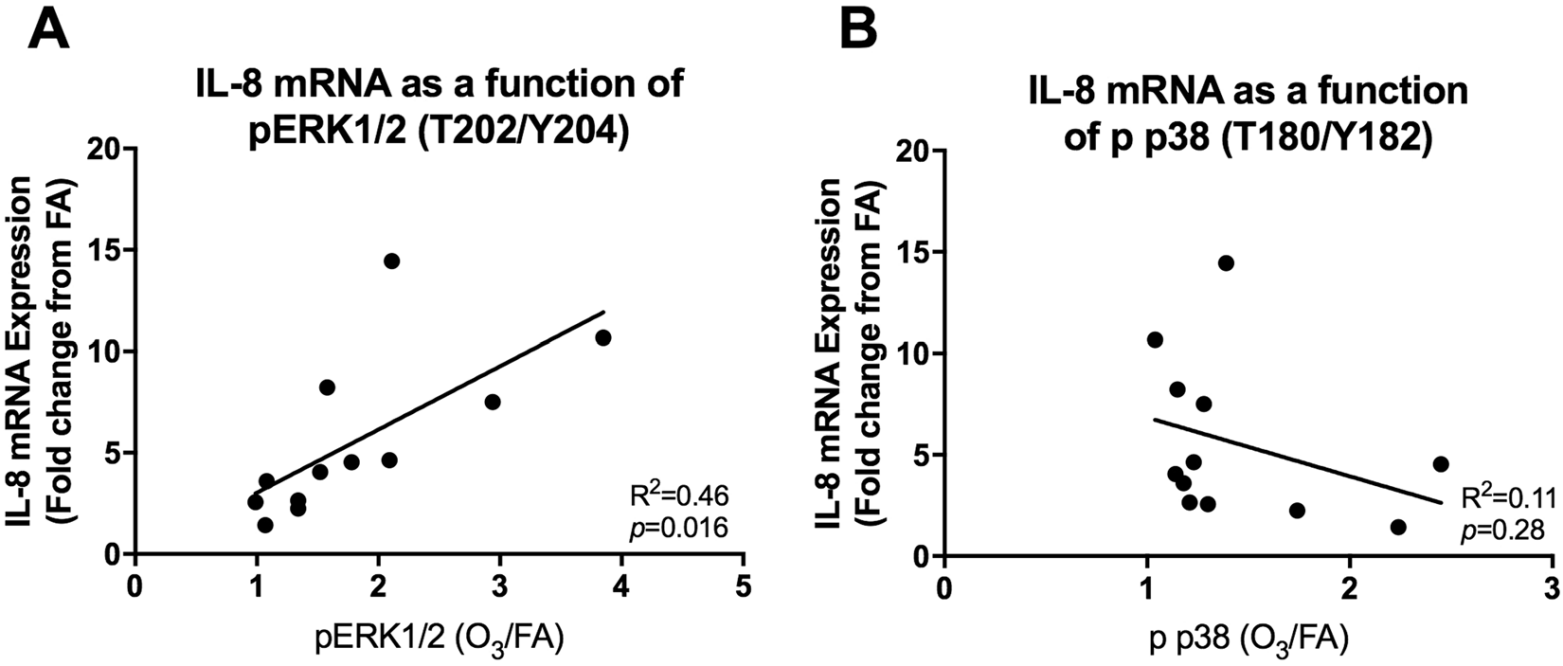
*IL-8* expression as a function of ERK1/2 and p38 activation. In addition to analyzing MAPK activation based on and high and low responder status, we analyzed the data in continuous format in which the ratio of activated protein (X-axis) was plotted against ozone mediated *IL-8* induction. This comparison was made for both ERK1/2 (**A**) as well as p38 (**B**). Figures were created using the same donors as depicted in Fig. 4 (n = 12; 6 high and 6 low responders).

In addition to comparing MAPK pathway activation in high and low *IL-8* responding cultures, we also compared these two variables in a continuous format via linear regression (Fig. 5). We found that *IL-8* expression was significantly correlated with changes in ERK1/2 phosphorylation (Fig. 5A; R^2^ = 0.46, *p* = 0.016), but not p38 phosphorylation (Fig. 5B; R^2^ = 0.11, *p* = 0.28).

### NFκB Activation

**Figure 6:**
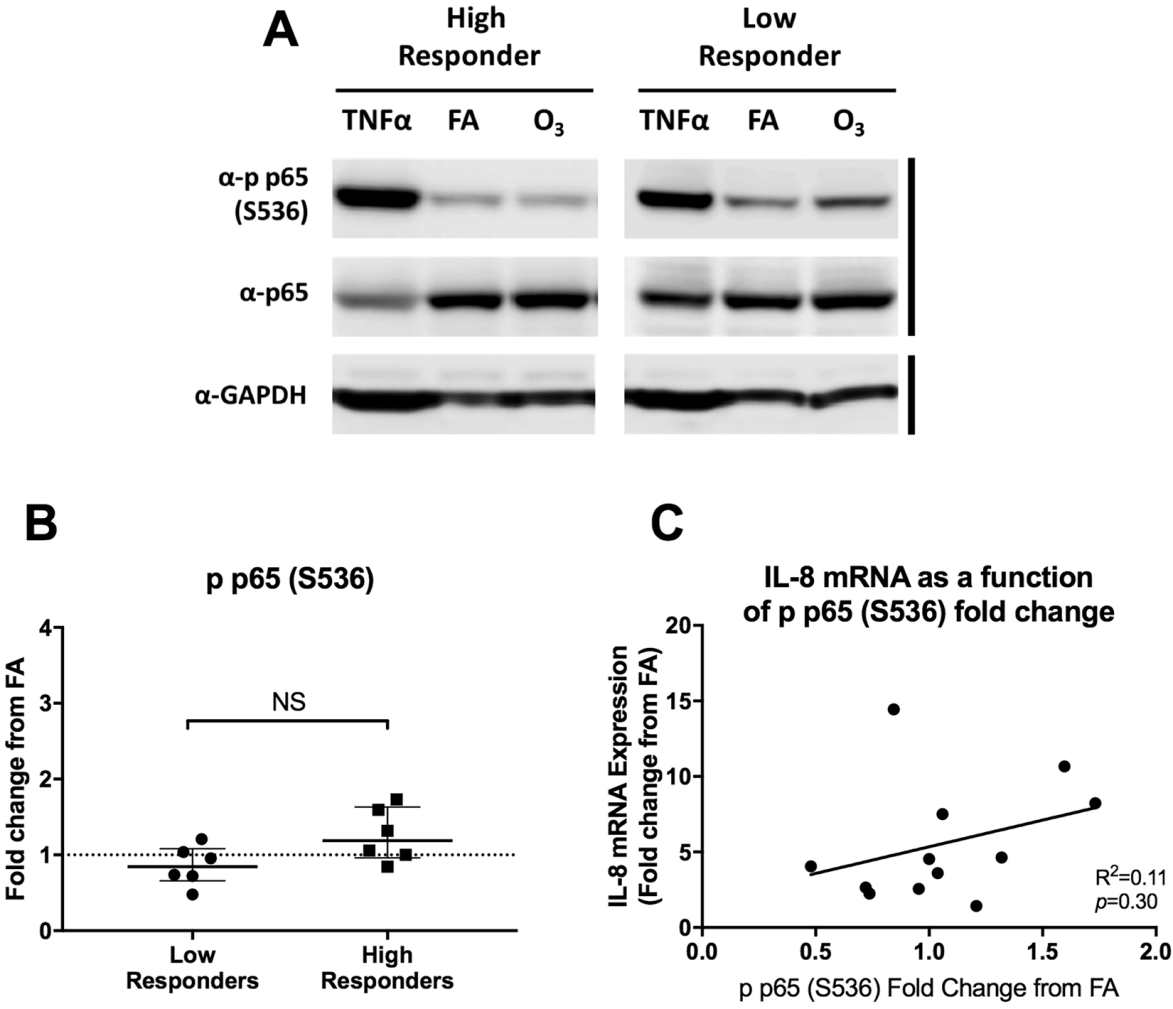
Comparison of p65 activation in high and low-responding cultures. (**A**) Using the same panel of donors as Fig. 4, we examined the p65 phosphorylation at S536, an indicator of canonical NFκB activation. Primary cells were stimulated with 10 ng/mL TNFα for 20 minutes as a positive control. Representative donors shown are the same donors depicted in Fig. 4. Both sets of blots were run on the same gel, immunoblotted, and imaged at the same time. **(B)** Median and interquartile range of ozone-induced phosphorylation changes across 6 high and 6 low responders (n = 12) as calculated by densitometric comparison to filtered air controls. No significant difference was found between these two groups (*p* = 0.07). **(C)** Linear regression showing the correlation between ozone-associated changes in p65 phosphorylation and *IL-8* mRNA expression.

In addition to p38 and ERK1/2 phosphorylation, we also compared the phosphorylation of the p65 NFκB subunit with ozone responsiveness (Fig. 6). Previous studies have demonstrated that the NFκB pathway is not as influential in ozone-mediated *IL-8* signaling in polarized phBECs^13^; however, given its importance in cell lines, we wanted to confirm these findings in differentiated phBECs. We found that p65 phosphorylation did not significantly differ between these groups, with low responders having a mean of 0.86 ± 0.26 and high responders a mean of 1.26 ± 0.35 (*p* = 0.07).

## Discussion

The mechanisms governing inter-individual variability in biological responses remain elusive despite wide spread observation. This issue is especially pertinent in the air pollution field, where extensive inter-variability complicates the prediction of exposure health effects and the identification of susceptible individuals. The air pollutant ozone can be used to model this variability, as ozone-mediated inflammatory responses vary widely between individuals, yet are highly reproducible within an individual. Here we show that an important source of ozone response variability may be differences in how airway epithelial cells respond to pollutant exposure via the transcription of pro-inflammatory mediators such as IL-8. Our data suggest that the level of *IL-8* transcription in response to ozone exposure may be an intrinsic property of airway epithelial cells that is modulated by the cooperative activity of two MAPKs, ERK1/2 and p38. This finding may provide important mechanstic information regarding pro-inflammatory response variability across environmental exposures and disease.

Respiratory epithelial cells play a key role in eliciting inflammatory responses, thus examining their biology may offer unique insights into the factors underlying variability in response to ozone exposure. We observed that ozone-mediated *IL-8* expression varied across phBEC cultures from different donors; however, cells can be sensitive to slight variations in culture environment^15^. Thus, we questioned whether the transcriptional variability we observed in the phBEC system was the result of intrinsic cellular properties or ‘noise.’ We confirmed that *IL-8* responses to ozone exposure could be recapitulated in phBECs collected from the same donors and cultured independently, suggesting that epithelial cell responses are intrinsic and that these ‘programs’ drive phBEC response variability. This novel finding supports the growing use of primary cells in environmental health research, as it suggests that cells collected from various donors can be used to investigate inter-individual variability. While our data suggest that epithelial cell responses are conserved in a donor-specific fashion, the basis of this ‘programming’ remains to be determined. While it may simply reflect genetic variability, it is possible that epithelial cells may retain an epigenetic imprint of their donor’s life history that can determine future responsiveness^16^. Although the factors that ultimately determine ozone-associated *IL-8* induction in epithelial cells are unclear, our findings suggest that the differential activation of MAPKs is likely a part of this programming.

Our data suggest that ozone-mediated *IL-8* expression is modulated by the activity of both the p38 and ERK1/2 pathways. While ERK1/2 mediates many functions related to proliferation and cell survival, p38 is more commonly associated with stress response and apoptosis. While these kinases have seemingly disparate roles, there is substantial crosstalk between these pathways and their coordinate activity is involved in many cellular functions. Here we report that the response to the pro-inflammatory stimulus ozone is one such function. This is evidenced by the fact that the most substantial decline in ozone-mediated *IL-8* expression is achieved when both kinases are simultaneously inhibited. These findings recapitulate recent results from our lab using an ozone exposure model and an alternative phBEC system^13^. Similar findings have also been reported in other model systems using MAPK inhibitors; for example, cytokine expression from the e*x vivo* stimulation of human alveolar macrophages with lipopolysaccharide (LPS) is partially reduced with the addition of either a p38 or ERK1/2 inhibitor, but the addition of both inhibitors is necessary to achieve a near abrogation of expression^17^. The same observation was noted during LPS stimulation of the monocyte cell line THP-1^18^. This consistent pattern may be explained by the fact that there is substantial cross talk between these pathways, where the inhibition of one pathway can result in the other becoming activated in compensation^19–21^. MAPK cross-activation is also a mechanism of chemotherapy resistance in cancer cells and is currently a major obstacle in cancer treatment^22^. This phenomenon can be observed in the Western Blot in Fig. 4, where the inhibition of p38 led to increased phosphorylation of ERK1/2 and RSK.

**Figure 7:**
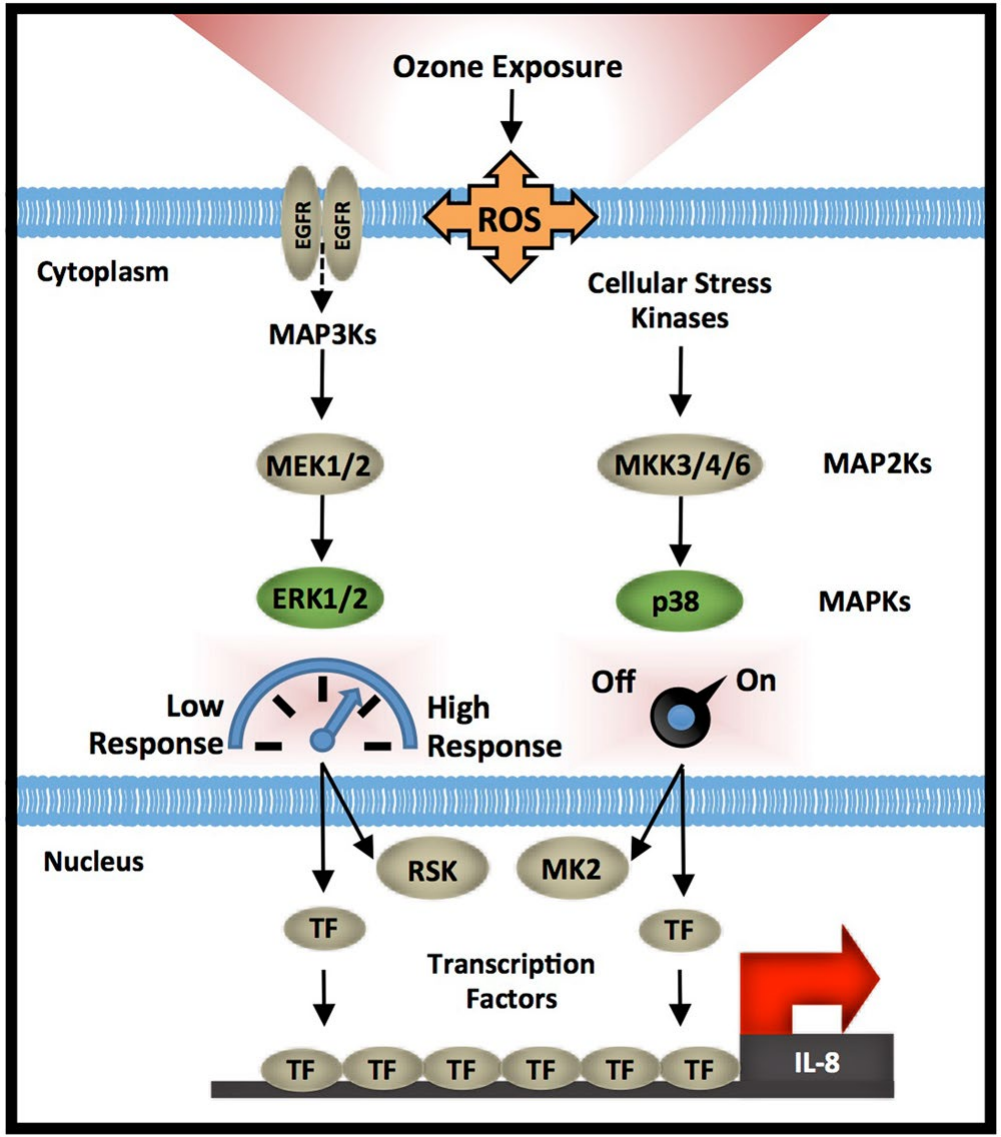
Paradigm describing how differences in MAPK signaling lead to inter-individual variability in phBEC ozone-mediated *IL-8* induction. Previous studies have suggested that ozone exposure leads to the activation of the MAP Kinases ERK1/2 and p38 by various mechanisms including the activation of the EGFR receptor. The activation of both kinases is important for full ozone-mediated *IL-8* induction, yet the magnitude of ozone-induced *IL-8* expression is only correlated with the level of ERK1/2 activation. Based on these observations, we propose a ‘dimmer switch’ model describing how p38 and ERK1/2 act in concert to regulate *IL-8* expression. A basal level of p38 activation is required to achieve full *IL-8* expression (acting as an ‘on/off’ switch), while the level of ERK1/2 pathway activation modulates the magnitude of expression (the ‘dimmer’). These kinases both activate effector proteins and kinases such as RSK and MK2, in addition to other transcription factors and stress-associated proteins. These ultimately converge on the *IL-8* promoter and regulate gene transcription.

Although p38 (but not ERK1/2) inhibition alone reduced *IL-8* expression, p38 phosphorylation was not correlated with *IL-8* expression across phBEC donors. Instead, *IL-8* expression was directly correlated to the magnitude of ERK1/2 activation. These observations led us to propose a ‘dimmer switch’ model explaining how p38 and ERK1/2 act together to modulate ozone-mediated *IL-8* expression (Fig. 7). In this model, a basal level of p38 activation is required for full *IL-8* induction (the on/off switch), but the level of ERK1/2 activation ultimately determines the magnitude of *IL-8* expression (the ‘dimmer’). The requirement for the activation of both kinases for full induction could be explained by the fact that p38 has an essential role in the stabilization of *IL-8* mRNA. In unstimulated cells, the 3′ UTR of *IL-8* mRNA contains several AUUUA motifs which confer instability and result in its rapid degradation; however, p38 plays an important role in stabilizing *IL-8* mRNA and preventing degradation^23,24^. This model can be used to further the understanding of molecular factors driving variability in the pro-inflammatory response to ozone.

This research raises important questions regarding how the interaction of these kinases modulates *IL-8* expression, and why ERK1/2 is differentially activated in high and low-responding cultures. Ozone is known to react with the airway surface and generate oxidation products and ROS^25^. These products, in turn, may directly or indirectly activate effectors upstream of ERK1/2. Such proposed effectors include the activation of the EGFR receptor, via the alteration of cell membrane lipid raft composition^26^. Alternatively, EGFR and other MAPKs may become phosphorylated as a result of ROS directly deactivating phosphatases, which typically act as a brake on MAPK activation^27,28^. Another proposed mechanism is that ozone exposure results in TLR4 signaling, which results in the activation of heat shock proteins, which in turn activate MAPKs^29^. Thus, activation of the ERK1/2 pathway may vary between high and low responders *in vitro* due to any number of differences in these effectors and pathways.

In addition to MAPKs, there are other important molecular factors likely shaping *IL-8* transcriptional response. This is suggested by the fact that even when both p38 and ERK1/2 inhibitors were used, we did not achieve a complete reduction of expression; moreover, *IL-8* inductions in low responders were not influenced by kinase inhibition. This residual *IL-8* induction may be due to low-level inputs from other cellular effectors, such as NRF2^30^.

Despite the novelty of these findings, this study is not without limitations. Our use of inhibitors was essential in this investigation, as fully differentiated phBECs are recalcitrant to transfection/transduction; yet off-target effects are always possible when using these compounds. We attempted to minimize this possibility by using SCH772984 and LY2228820, which are novel compounds noted for their specificity and efficacy even in nanomolar concentrations^31,32^. Here we use a phBEC model to examine molecular mechanisms underlying differences in *IL-8* expression. While our use of phBEC allowed us to model epithelial cell responses across many individuals, inflammatory responses *in vivo* involve complex interactions with many different cell types. Future studies could utilize co-culture techniques to assess these relationships and whether similar patterns of ERK1/2 activation are observed in other cell types, such as neutrophils, macrophages, fibroblasts, etc. Despite the fact that relative to many other *in vitro* toxicity studies we have a large number of primary cell donors, we still have too few donors to sufficiently power a study examining the influence of characteristics such as genotype, age, ethnicity, etc. This study also has some unique strengths: whereas previous studies have relied heavily on cell lines, we have used the phBEC system which is more physiologically relevant and facilitates the exploration of inter-individual variability, which formerly was only possible using *in vivo* exposure studies.

Epithelial cells exposed to ozone exhibit inter-individual variability in *IL-8* transcriptional responses, which may contribute to the inter-individual variability in inflammatory response observed in human clinical ozone exposure studies. The level of ozone-induced *IL-8* transcription is reproducible in phBECs from the same individuals even when collected and cultured at different times, suggesting that ozone-associated *IL-8* production is an intrinsic property of epithelial cells. As a first step to describing the molecular mechanisms underlying these differences, we propose a model where p38 and ERK1/2 activation modulate the induction of the pro-inflammatory cytokine *IL-8*. Because MAPK signaling is highly integrated with many other signaling networks, it is possible that other previously described ozone susceptibility factors could exert their effects by modulating MAPK signaling. Thus, our model could be used to synthesize previous work and streamline the understanding of susceptibility to ozone as well as many other pollutants.

## Methods

### *In vitro* epithelial cell ozone responses: Primary cell collection, culture, and ozone exposure

Primary human bronchial epithelial cells were obtained via bronchial brushing from healthy non-smokers with no more than a 1-pack year smoking history. Donors gave their informed consent after being informed of risks and procedures. The consent and collection protocol were approved by the UNC School of Medicine Committee on the Protection of the Rights of Human Subjects and by the US EPA. All experiments and procedures were performed in accordance with relevant guidelines and regulations from these institutions. To determine if phBECs from the same donor had consistent responses to ozone over time we compared *IL-8* inductions between cells from the same donor that were collected during two different bronchoscopies. Cells were cultured and differentiated as described by Ross *et al*.^14^. Briefly, cells were expanded for three passages and then plated on 24 mm Transwell inserts with 0.4 µm pores (Corning Life Sciences, Tewksbury, MA). Three inserts per treatment per donor were used. Once confluent, cells were submerged for 48 hours with 500 nM retinoic acid. Afterward the apical layer of medium was removed creating an air-liquid interface (ALI). Cells were maintained for 24 days at ALI and supplemented with 100 nM retinoic acid to promote differentiation into a pseudostratified columnar epithelium. Prior to each ozone exposure, the basolateral medium was replaced and the apical surface was washed with 500 µL Dulbecco’s PBS (Life Technologies, Carlsbad, CA) to remove cellular debris and secretions. After a two-hour acclimation period cells were exposed to a filtered air (FA) control or 0.5 ppm ozone for two hours. This dose is conventionally used *in vitro* and is similar to doses used in clinical ozone exposure studies^33^. Immediately following exposure cells designated for MAPK analysis were harvested as described below. Two hours following exposure, cells designated for gene expression analysis were harvested in RNA lysis buffer (Life Technologies) and stored at −80 °C until they were ready for processing. As a positive control for NFκB pathway activation, primary cells were stimulated with 10 ng/mL TNFα for 20 minutes.

### RT-qPCR

We used *IL-8* transcription as our primary read-out because it is more closely linked to cellular signaling relative to protein levels, which may be influenced by further regulatory processes such as degradation. RNA was extracted from lysed samples using an RNA Mini Kit (Life Technologies) and quantified using a Nanodrop ND1000. One (1) μg was used to synthesize cDNA using iScript Reverse Transcription Kits (Bio-Rad, Hercules, CA). Gene expression was assessed in technical triplicates using TaqMan RT-qPCR primers and probes (Supplementary Methods) and the CFX96 qPCR system (Bio-Rad). Target gene expression was normalized to β-actin (ACTB) and then expressed as a fold changes between filtered air and ozone exposure treatments using the Pfaffl method^34^.

### MAPK Inhibition

To investigate the role of MAPK activity in ozone-associated *IL-8* induction, the ERK1/2 and p38 kinase inhibitors SCH772984 and LY2228820 (Cayman Chemical, Ann Arbor, MI), respectively, were added to cell media to a final concentration of 250 nM with 0.2% DMSO during media change two hours prior to ozone exposure. These remained in the media throughout exposure until harvest. While SCH772984 prevents ERK1/2 phosphorylation and inhibits its kinase activity, LY2228820 inhibits the kinase activity of p38 but does not prevent it from being phosphorylated^31,32^. Thus, to verify ERK1/2 and p38 kinase activity inhibition, we examined the phosphorylation of the downstream targets ribosomal s6 kinase (RSK) and mitogen activated protein kinase-activated protein kinase 2 (MK2), respectively via Western blotting. As a positive control for p38 and ERK1/2 activation, primary cells were stimulated with media that contained 5% FBS and 5 μg/mL LPS for four hours.

### MAPK Pathway Analysis

We compared ozone-associated MAPK activation between donors that had high versus low *IL-8* inductions by examining phosphorylation at specific residues. Cellular extracts were prepared in RIPA buffer (50 mM Tris, pH 8.0; 150 mM NaCl; 1% Triton X-100; 400 μM EDTA; 10% glycerol; 0.1% SDS; 0.1% deoxycholate) with protease (cOmplete EDTA-free, Roche, Indianapolis, IN) and phosphatase (PhosSTOP, Roche) inhibitors. Cellular debris was then removed via centrifugation and aliquots of RIPA extract were removed for protein quantification via BCA assay (ThermoFisher, Waltham, MA). The remaining supernatant was supplemented with Laemmli buffer to a final concentration of 1× (60 mM Tris, pH 6.8; 200 mM DTT; 10% glycerol; 2% SDS; 0.05% bromophenol blue), incubated at 95 °C for five minutes, aliquoted, and stored at −80 °C. For each sample, equal amounts of protein were loaded into SDS-PAGE gels, electrophoresed, and transferred to nitrocellulose membranes (Bio-Rad) via tank transfer. Following primary antibody binding, horseradish peroxidase (HRP)-conjugated secondary antibodies and Pierce Enhanced Chemiluminescence (ECL) Western blotting substrate (ThermoFisher) were used to generate chemiluminescence. Immunoblots were imaged on a LAS-3000 detection system (Fuji/GE Healthcare, Pittsburgh, PA). Densitometry analysis was performed using ImageJ software (National Institutes of Health, Bethesda, MD). The pixel density from the phosphorylated protein was normalized to the pixel density from total protein levels, after which the fold changes between treated and filtered air conditions were calculated. The antibodies used for this analysis were all obtained from Cell Signaling Technologies (Danvers, MA, USA): GAPDH (#5174), α-ERK1/2 (#4695), α-ERK1/2 (T202/Y204)p (#4370), α-MAPKAPK-2 (Thr334)p (#3007), α-MEK1/2 (S217/221)p (#9121), α-MEK1/2 (#9126), α-MKK4/SEK1 (S257/T261)p (#9156), α-MKK4/SEK1 (#9152), α-p38 (#9212), α-p38 (T180/Y182)p (#4511), α-p65 (S536)p (#3033), α-p65 (#8242).

### Statistical Analysis

All statistical analyses were conducted using GraphPad Prism 6.07 (GraphPad Software, La Jolla, California, USA). For all analyses a *p*-value of less than 0.05 was considered statistically significant. Simple linear regression was used to relate *IL-8* responses between repeated bronchoscopies and exposures. To determine if inhibitor treatments significantly reduced *IL-8* induction from the ozone-vehicle (O_3_−V) control, a two-way ANOVA with Dunnett’s Multiple Comparisons was used. Kinase phosphorylation in high and low responders was compared using a non-parametric Mann-Whitney test. Linear regression was used to compare *IL-8* mRNA expression with changes in the phosphorylation of ERK1/2, p38, and p65.

## Electronic supplementary material

Figure S1
