# Supplementary material for: ERK1/2 and p38 regulate inter-individual variability in ozone-mediated IL-8 gene expression in primary human bronchial epithelial cells
Source: Sci Rep. 2018 Jun 20;8:9398. doi: 10.1038/s41598-018-27662-0 (PMC6010411; doi:10.1038/s41598-018-27662-0)
Supplement: Supplementary file 1 — Figure S1 [file 41598_2018_27662_MOESM1_ESM.pdf]

ERK1/2 and p38 regulate inter-individual variability in ozone-mediated *IL-8* gene expression in primary human bronchial epithelial cells

Emma C. Bowers‡, Shaun D. McCullough†, David S. Morgan†, Lisa A. Dailey†, and David Diaz-Sanchez†*

‡Curriculum in Toxicology, University of North Carolina – Chapel Hill, Chapel Hill, NC, 27599
†Environmental Public Health Division, National Health and Environmental Effects Research Laboratory, U.S. Environmental Protection Agency, Research Triangle Park, NC 27711

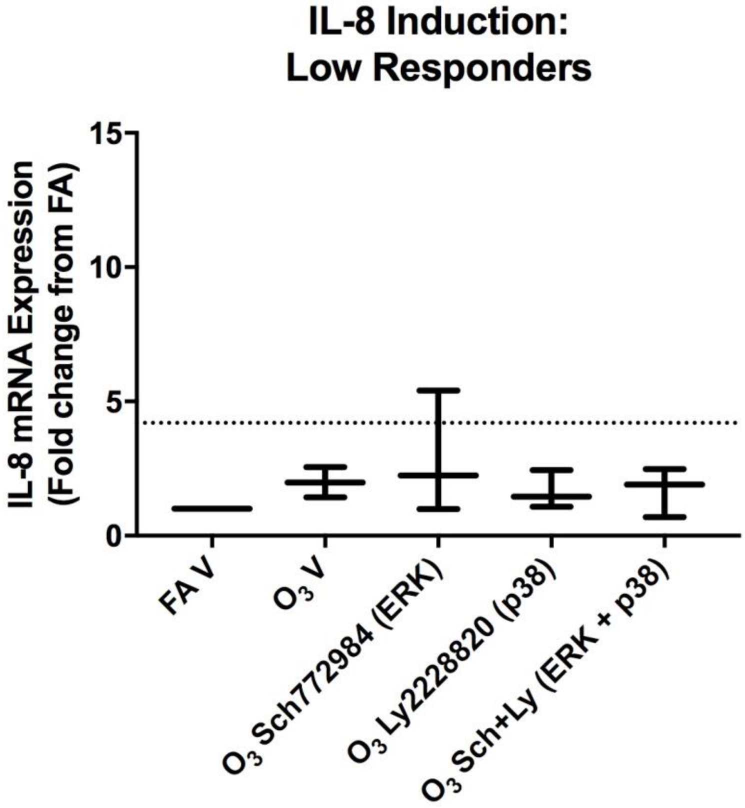

Figure S1. The influence of ERK and p38 inhibition on the ozone-associated *IL-8* expression in low responders. To determine whether the activation of the MAP kinases ERK1/2 and p38 were required for ozone-mediated *IL-8* induction, inhibitors of these kinases (SCH772984 and LY2228820, respectively) were added to cell media two hours prior to ozone exposure. Seven donors were used, three low responders (Figure S1) and four high responders (Figure 3). *IL-8* inductions were normalized to a filtered air vehicle control. Mean *IL-8* inductions were compared between the ozone-vehicle control (O_3-V) and all drug treatments via 2-way ANOVA.
